# Characterization of a Leptin Receptor Paralog and Its Response to Fasting in Rainbow Trout (*Oncorhynchus mykiss*)

**DOI:** 10.3390/ijms22147732

**Published:** 2021-07-20

**Authors:** Jamie L. Mankiewicz, Beth M. Cleveland

**Affiliations:** National Center for Cool and Cold Water Aquaculture, Agricultural Research Service, United States Department of Agriculture, Kearneysville, WV 25430, USA; jamie.mankiewicz@usda.gov

**Keywords:** leptin receptor, rainbow trout, duplicate, paralog, tissue distribution, fasting

## Abstract

Leptin is a cytokine that regulates appetite and energy expenditure, where in fishes it is primarily produced in the liver and acts to mobilize carbohydrates. Most fishes have only one leptin receptor (LepR/LepRA1), however, paralogs have recently been documented in a few species. Here we reveal a second leptin receptor (LepRA2) in rainbow trout that is 77% similar to trout LepRA1. Phylogenetic analyses show a salmonid specific genome duplication event as the probable origin of the second LepR in trout. Tissues distributions showed tissue specific expression of these receptors, with *lepra1* highest in the ovaries, nearly 50-fold higher than *lepra2*. Interestingly, *lepra2* was most highly expressed in the liver while hepatic *lepra1* levels were low. Feed deprivation elicited a decline in plasma leptin, an increase in hepatic *lepra2* by one week and remained elevated at two weeks, while liver expression of *lepra1* remained low. By contrast, muscle *lepra1* mRNA increased at one and two weeks of fasting, while adipose *lepra1* was concordantly lower in fasted fish. *lepra2* transcript levels were not affected in muscle and fat. These data show *lepra1* and *lepra2* are differentially expressed across tissues and during feed deprivation, suggesting paralog- and tissue-specific functions for these leptin receptors.

## 1. Introduction

The hormone leptin has been well characterized for its role in regulating appetite as an anorexigen in vertebrates and it has also been implicated in many other processes including immunity, reproduction, energy homeostasis, and hydromineral balance (reviewed in [1,2,3]). Leptin is a 16 kDa class-I helical cytokine encoded by the obese (*ob*) gene in mammals, where it acts as an adipostat and circulates in proportions relative to adipose tissue and increases postprandially [4,5]. By contrast, in fishes and other ectotherms, leptin is primarily produced in the liver where it appears to focus on mobilizing carbohydrates instead of lipids [3]. Leptin expression is highly sensitive to glycemia in vivo (Mozambique tilapia, [6]) and in vitro in hepatocytes (grass carp, [7]), further implicating the hormone in carbohydrate metabolism. There have been wide ranging responses of leptin to fasting reported in fishes (reviewed in [8]) ranging from increases (rainbow trout, [9]; Mozambique tilapia, [10]), decreases (hybrid striped bass, [11]; red-bellied piranha, [12]), and no response of leptin to feed deprivation (goldfish, [13]). There are two distinct leptin proteins present in most fishes: leptin A (LepA) and leptin B (LepB), while salmonids and cyprinids have four paralogs including two LepA’s (LepA1 and LepA2) and two LepB’s (LepB1 and LepB2) (reviewed in [14]). However, some salmonids such as Arctic char (*Salvelinus alpinus*) and rainbow trout (*Oncorhynchus mykiss*) appear to only have preserved the LepB1 variant [15]. To date, most studies have focused on the dominant form, LepA, as mRNA levels can range from 10–100 times higher than LepB [3,16].

Leptin elicits its actions through a class-I cytokine receptor characterized by cysteine residues [17]. Leptin receptor (LepR) homodimerizes at the cell membrane which transduces into intracellular signaling controlled through a highly conserved JAK/STAT pathway [18,19,20,21,22]. There are numerous splice variants of LepR that have been identified in mammals which have been categorized as short, long, and secreted forms [17]. Versions of alternatively spliced leptin receptors have been characterized in some teleost fish including crucian carp (*Carassius carassius*; [23]), European sea bass (*Dicentrarchus labrax*; [24]), Atlantic salmon (*Salmo salar*; [25]), and rainbow trout (*O. mykiss*; [26]), where similar to mammals the long form is the full functional LepR. Furthermore, three short form LepR variants that act as binding proteins and are comprised of only the extracellular segment were identified in rainbow trout [26]. Interestingly, all of the alternatively spliced LepR variants had different tissue expression patterns and were regulated by the physiological state of the fish [26]. As with leptin, most teleost LepRs share a low level of amino acid sequence identity with that of the mammalian LepR, although there remains high conservation in tertiary structure and ligand binding [27]. The leptin receptor is ubiquitously expressed across tissues, however, high abundances have been reported in gonadal tissue and the brain, specifically the hypothalamus and pituitary, further linking this region to leptin control of food intake [3].

About 350 million years ago, there was a 3R genome duplication event which was the cause of a whole genome duplication specific to teleost fish [28]. This event was the likely source of the leptin B paralog appearing in fishes [15,16]. Interestingly, in nearly all vertebrates there has only been a single leptin receptor identified, including for most teleost fish where many genes have second copies and produce functional proteins from duplication [28,29]. However, recently a second leptin receptor has been identified in a few species of fish: the Asian arowana (LepRa/LepRb, *Scleropages formosus*; reviewed in [16]), two species of eel (LepRa/LepRb, European eel, *Anguilla anguilla* and Japanese eel, *Anguilla japonica*; [29]), and Atlantic salmon (LepRA1/LepRA2, *S. salar*; [30]). It is unclear if the duplicated eel and arowana LepRs were present in teleost ancestors and then lost, but it appears to be from an ancient duplication event [16,29]. By contrast, more recently there was a 4R salmonid specific genome duplication event that has been dated to only 25 to 100 million years ago and is likely the reason for the duplicated LepA and LepB variants in some salmonids and for the presence of a second receptor in Atlantic salmon [30,31]. In rainbow trout, the functional long-form leptin receptor has been characterized (LepRL, and herein to distinguish between paralogs we use the same nomenclature as Atlantic salmon, LepRA1) [26,30,32]. With the aid of the rainbow trout genome (GenBank accession # GCA_013265735.3) and the sequence for LepRA2 from the closely related Atlantic salmon, we have also identified a second leptin receptor in *O. mykiss* (LepRA2).

As studies on leptin in ectothermic vertebrates advance, it is apparent that leptin plays a key role in metabolism and energy balance. It is important to understand the function of leptin and its receptors, especially in fishes where feeding behaviors and energy reserves can differ considerably from mammals. Rainbow trout is a high value aquaculture species and well-established as a genetic and physiological model. Here we characterize a LepRA2 paralog in rainbow trout by performing evolutionary analyses and tissue distributions of the receptors. We hypothesized that the two leptin receptors identified in rainbow trout may have divergent functions and may be differentially expressed under altered metabolic conditions where leptin may be regulated, such as a fasted or stressed state. To elucidate the roles of both leptin receptors under catabolic conditions we exposed the fish to a two-week period of feed deprivation.

## 2. Results

### 2.1. Phylogenetic Analyses and Sequence Alignments

**Figure 1:**
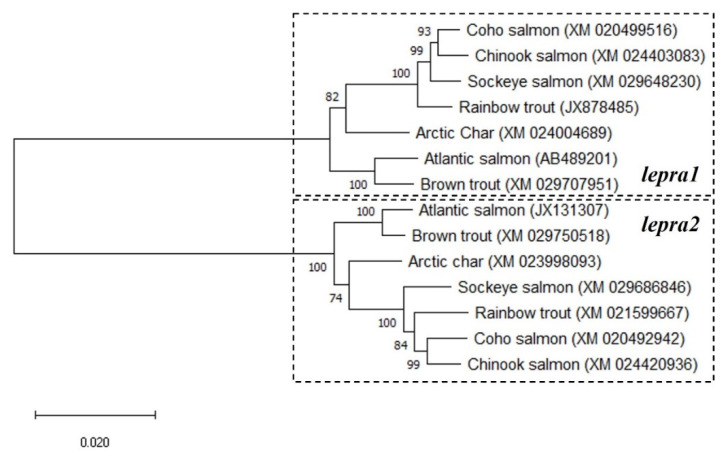
Evolutionary analysis of salmonid leptin receptor nucleotide sequences using the Maximum Likelihood method. The bootstrap values at nodes were based on 1000 replicates. This analysis involved 14 nucleotide sequences. GenBank accession #’s are in parenthesis. There were a total of 3540 positions in the final dataset. Branch lengths were measured in the number of substitutions per site. Evolutionary analyses were conducted in MEGA X.

The BLAST analyses using the *lepra1* and *lepra2* nucleotide sequences from Atlantic salmon (*S. salar*, [30]; AB489201 and JX131307, respectively) revealed six predicted leptin receptor-like sequences in other salmonids of high similarity. The receptors show 95–99% sequence identity to *S. salar lepra2* and 85–87% to the *lepra1* paralog of that species; rainbow trout (*O. mykiss*; JX878485 and XM_021599667), Arctic char (*S. alpinus*; XM_024004689 and XM_023998093), coho salmon (*Oncorhynchus kisutch*; XM_020499516 and XM_020492942), sockeye salmon (*Oncorhynchus nerka*; XM_029648230 and XM_029686846), chinook salmon (*Oncorhynchus tshawytscha;* XM_024403083 and XM_024420936), and brown trout (*Salmo trutta*; XM_029707951 and XM_029750518). When phylogenetic analyses were performed on the 14 salmonid leptin receptor nucleotide sequences, two distinct clades were formed, which were then identified for each *lepr* paralog (Figure 1).

**Figure 2:**
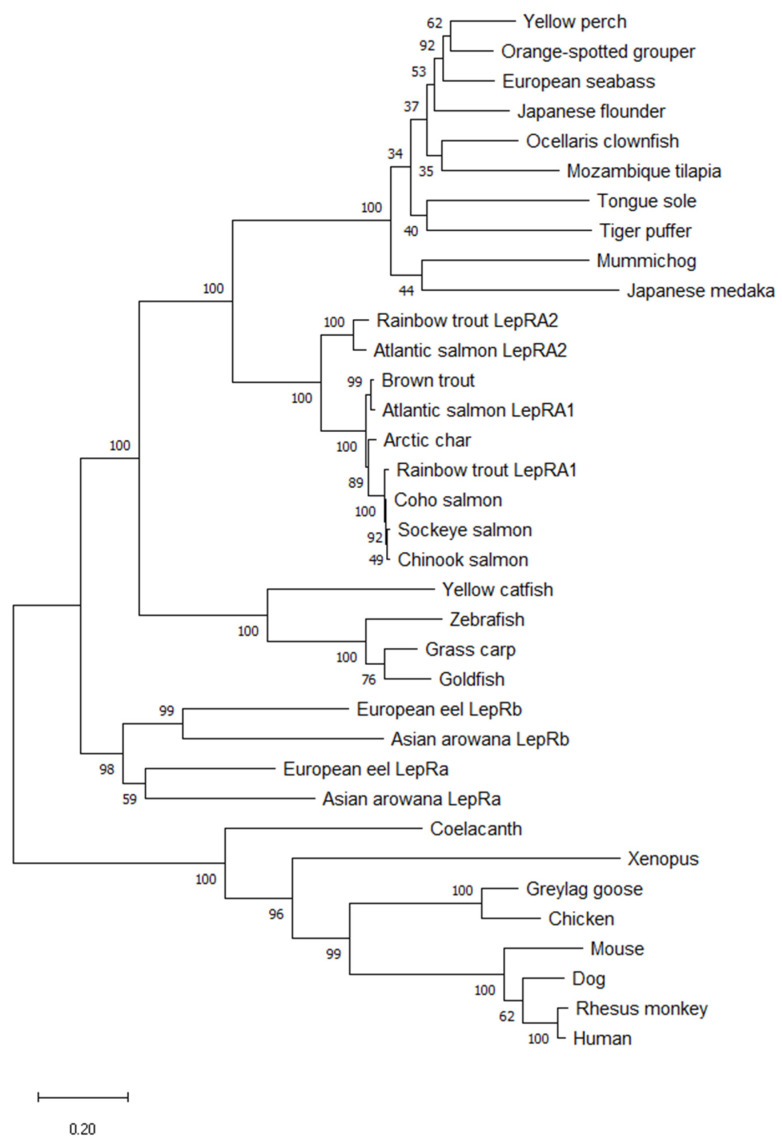
Evolutionary analysis of leptin receptor amino acid sequences using the Maximum Likelihood method. The bootstrap values at nodes were based on 1000 replicates. This analysis involved 35 amino acid sequences. There were a total of 1448 positions in the final dataset. Branch lengths were measured in the number of substitutions per site. Evolutionary analyses were conducted in MEGA X.

**Figure 3:**
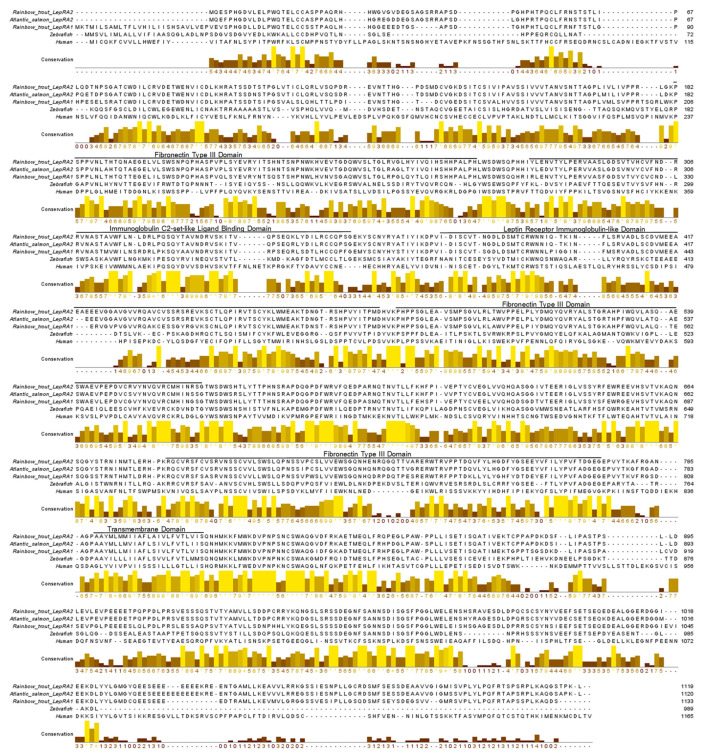
Amino acid sequence alignment of leptin receptors from rainbow trout (*Oncorhynchus mykiss*, LepRA1: XP_021460283; LepRA2: XP_021455342), Atlantic salmon (*Salmo salar*, LepRA2: NP_001315080), zebrafish (*Danio rerio*, AAY16198), and human (*Homo sapiens*, AAA93015). Predicted conserved functional domains for rainbow trout LepRA2 are labeled and indicated by black lines spanning the domain. The boxes below the sequence indicate areas of conservation. High conservation between sequences shows brighter color and higher score values, while low conservation is indicated with progressively darker color and decreased values.

The amino acid sequences for the duplicated LepR in Atlantic salmon (*S. salar*: BAI23197 and NP_001315080), rainbow trout (*O. mykiss*: XP_021460283 and XP_021455342), Asian arowana (*S. formosus*: XP_018609810 and KPP63040), European eel (*A. Anguilla*: CEE15397 and CEE15398, [29]), were analyzed along with 27 other LepR protein sequences including 5 salmonids (Arctic char: XP_023860456, coho salmon: XP_020355105, sockeye salmon: XP_029504090, chinook salmon: XP_024258849, and brown trout: XP_029563811), a range of 15 additional species of fishes (zebrafish: AAY16198, Japanese medaka: BAH24203, Mozambique tilapia: AGT28753, clown anemonefish: XP_023146279, tiger puffer: BAG67079, mummichog: JAR68227, orange-spotted grouper: AFU55262, European seabass: AIB06819, yellow perch: XP_028442652, yellow catfish: AFO67946, grass carp: AFU35431,tongue sole: ARX70259, Japanese flounder: XP_019953845, goldfish: XP_026120678, and coelacanth: XP_006009523), and 7 tetrapods represented by amphibian, mammals, and birds (clawed frog: XP_018116241, chicken: BAA94292, greylag goose: AEE61372, mouse: AAC52705, dog: NP_001019805, rhesus monkey: NP_001027991, and human: AAA93015). The phylogenetic tree generated using these 35 sequences exhibits initial branching for tetrapods and teleosts, with coelacanth LepR showing closer relation to tetrapods. The teleost node has branching for more ancient fishes (eel and arowana) and the rest of the teleosts split into different multiple clades; for cyprinids and catfish, for more highly derived teleosts (i.e., pufferfish, tilapia, etc.), and for salmonids (Figure 2). Where present, the duplicated leptin receptor paralogs cluster together in their respective clades. The amino acid sequence alignment of rainbow trout LepRA1 and LepRA2 shows high conservation between paralogs and with Atlantic salmon LepRA2 (Figure 3). Rainbow trout LepRA1 and LepRA2 paralogs share a 77.1% amino acid sequence identity, while trout LepRA2 is 92.8% similar to Atlantic salmon LepRA2. Primary sequence conservation with human LepR are low for both paralogs at 30.8% and 32.2% for trout LepRA1 and LepRA2, respectively. However, predicted receptor domains remain conserved (Leptin Receptor: IPR015752). Extracellular domains (amino acids 1–788) include 3 fibronectin type 3 domains (IPR003961; 182-266, 484-566, 683-773), one immunoglobulin C2-set-like ligand-binding domain (IPR010457; 284-357), and one LepR immunoglobulin-like domain (IPR041182; 376-480). The transmembrane domain spans amino acids 789-811, while the cytoplasmic, intracellular domain ranges from amino acids 812-1119.

### 2.2. Tissue Distribution

**Figure 4:**
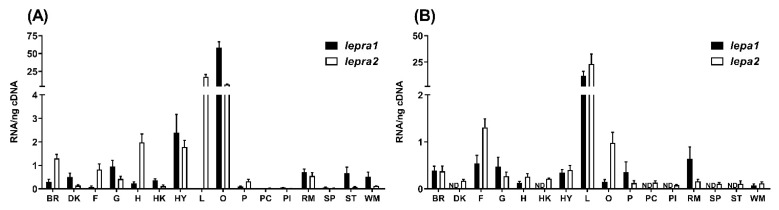
mRNA tissue distribution of (**A**) two leptin receptors, *lepra1* and *lepra2*, and two leptins (**B**) *lepa1* and *lepa2*, in adult female rainbow trout. Expression levels were determined by qPCR amplification in the brain (BR), distal kidney (DK), fat (F), gill (G), heart (H), head kidney (HK), hypothalamus (HY), liver (L), ovary (O), pituitary (P), pyloric caeca (PC), posterior intestine (PI), red muscle (RM), spleen (SP), stomach (ST), and white muscle (WM). ND = levels not detected. Values were normalized to the total nanograms of RNA used in cDNA synthesis and are expressed as the mean ± SEM (*n* = 5).

Abundance of mRNA levels of *lepra1*, *lepra2*, *lepa1*, and *lepa2* were analyzed across a variety of tissues by quantitative real-time PCR (qPCR). Both receptors were expressed across all tissues analyzed, however, there were some marked differences (Figure 4A).

*lepra1* was most highly expressed in the ovaries, at over 50× higher than *lepra2*. *lepra1* was expressed near 5× higher levels in the stomach and white muscle compared to *lepra2*. Interestingly, hepatic *lepra1* levels were low while *lepra2* was at levels over 17× higher. *lepa1* mRNA expression was highest in the liver with some lower levels in the brain, fat, gills, heart, hypothalamus, ovaries, pituitary, muscle, and no detectable expression in other tissues examined. Likewise, *lepa2* had the highest levels in the liver with low levels in most other tissues except fat and ovaries where transcript abundance was 2× and 7× higher than *lepa1* (Figure 4B).

### 2.3. Physiological Response to Feed Deprivation

**Figure 5:**
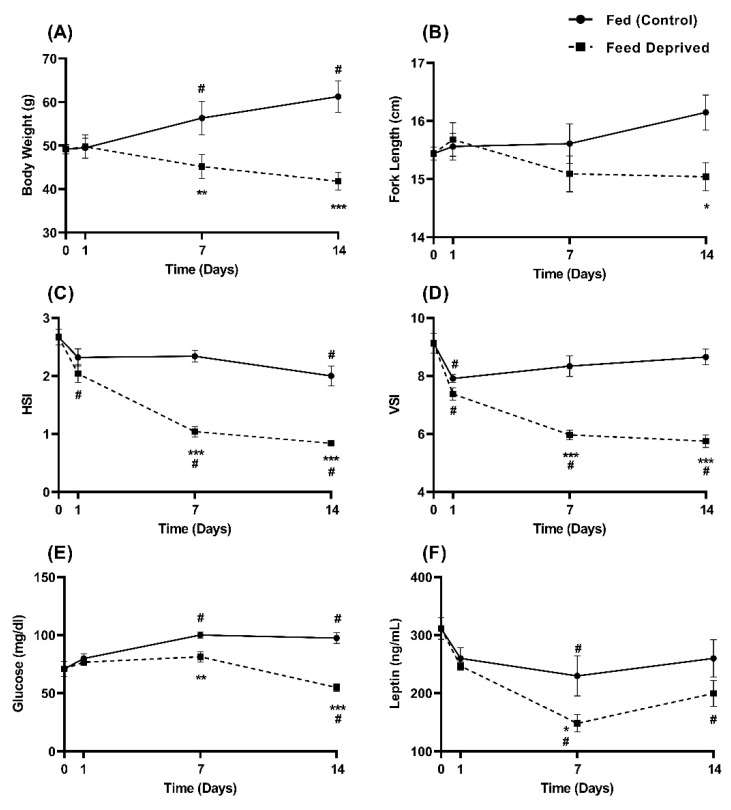
Rainbow trout were feed deprived for 14 days and compared to fed controls. (**A**) body weight (g), (**B**) fork length (cm), (**C**) blood glucose (mg/dL), (**D**) hepatosomatic index (HSI), (**E**) viserosomatic index (VSI), and (**F**) plasma leptin (ng/mL). Values reported as means ± SEM. # denote significant differences from time 0 within each treatment. * denote significant differences between treatments within each time point. (*n* = 9–12, except weight and length *n* = 16–84; * *p* ≤ 0.02, ** *p* ≤ 0.005, *** *p* ≤ 0.001).

There were no significant changes observed between fed and fasted fish after one day of feed deprivation (Figure 5). Rainbow trout that were exposed to one week of feed deprivation had significantly lower body weight relative to fed controls (Figure 5A; *p* = 0.001; fed: 56.31 ± 3.88 g, fasted: 45.17 ± 2.74 g), in addition to lower hepatosomatic index (HSI, Figure 5C; *p* < 0.001; fed: 2.34 ± 0.10%, fasted: 1.04 ± 0.09%), viscerosomatic index (VSI, Figure 5D; *p* < 0.001; fed: 8.34 ± 0.36%, fasted: 5.97 ± 0.17%), blood glucose (Figure 5E; *p* = 0.005; fed: 100.08 ± 2.78 mg/dL, fasted: 81.33 ± 4.48 mg/dL), and plasma leptin (Figure 5F; *p* < 0.01; fed: 229.56 ± 34.32 ng/mL, fasted: 148.22 ± 14.85 ng/mL). Fork length was not significantly different with one week of fasting (Figure 5B; *p* = 0.14; fed: 15.61 ± 0.34 cm, fasted: 15.09 ± 0.31 cm). After two weeks of feed deprivation, fasted fish had significantly lower body weight relative to fed controls (*p* < 0.001; fed: 61.26 ± 3.65 g, fasted: 41.81 ± 2.01 g), fork length (*p* = 0.02; fed: 16.15 ± 0.30 cm, fasted: 15.04 ± 0.24 cm), blood glucose (*p* < 0.001; fed: 97.42 ± 4.62 mg/dL, fasted: 54.83 ± 3.30 mg/dL), HSI (*p* < 0.001; fed: 2.00 ± 0.17%, fasted: 0.84 ± 0.05%), and VSI (*p* < 0.001; fed: 8.65 ± 0.27%, fasted: 5.75 ± 0.22%). Plasma leptin was numerically lower in feed deprived fish at two weeks, albeit not significant (*p* = 0.06; fed: 259.99 ± 32.25 ng/mL, fasted: 199.39 ± 22.51 ng/mL).

### 2.4. Response of Leptin and Leptin Receptor Expression to Feed Deprivation

**Figure 6:**
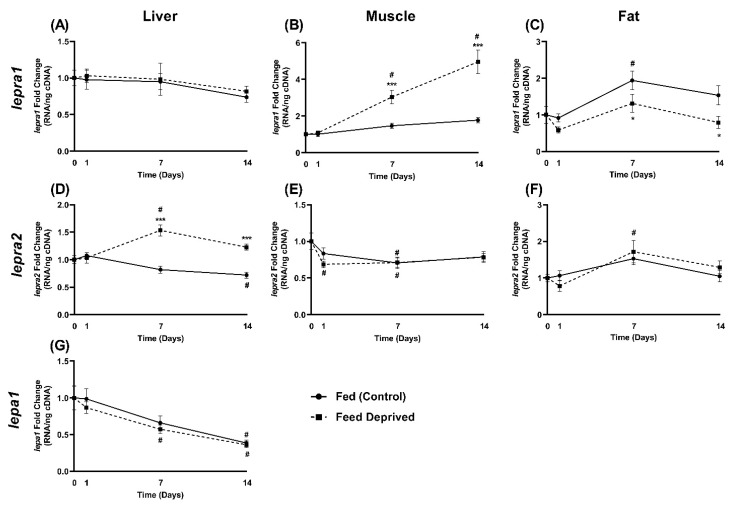
Rainbow trout were feed deprived for 14 days and compared to fed controls. mRNA levels of *lepra1* in (**A**) liver, (**B**) white muscle, (**C**) fat, and levels of *lepra2* in (**D**) liver, (**E**) white muscle, (**F**) fat, and *lepa1* in (**G**) liver, were determined by qPCR. Values were normalized to the total nanograms of RNA used in cDNA synthesis and are expressed as the mean fold change from the baseline at T = 0 ± SEM (*n* = 9–12, * *p* ≤ 0.04, *** *p* ≤ 0.001). # denote significant differences from time 0 within each treatment. * denote significant differences between treatments within each time point.

White muscle, liver, and fat tissues were analyzed for mRNA levels of *lepra1* and *lepra2* in response to fasting (Figure 6). No changes were detected in any tissue within one day of feed deprivation. Muscle *lepra1* mRNA levels increased significantly with fasting at one week (*p* < 0.001; fed: 1.47 ± 0.14, fasted: 3.04 ± 0.36), and by almost 3-fold at two weeks of feed deprivation (*p* < 0.001; fed: 1.78 ± 0.16, fasted: 4.96 ± 0.64). Interestingly, *lepra1* transcript levels in fat were significantly lower than fed fish at one week (*p* = 0.04; fed: 1.94 ± 0.26, fasted: 1.32 ± 0.24) and two weeks (*p* = 0.02; fed: 1.53 ± 0.26, fasted: 0.79 ± 0.16). No significant differences from fed controls were detected for *lepra2* in muscle or fat tissue. Liver *lepra1* mRNA was low and no changes were observed with fasting, however, *lepra2* levels were elevated near 2-fold compared to fed fish at one week (*p* < 0.001; fed: 0.82 ± 0.06, fasted: 1.53 ± 0.10) and two weeks (*p* < 0.001; fed: 0.72 ± 0.05, fasted: 1.23 ± 0.05). No changes in hepatic *lepa1* levels were detected with feed deprivation (Figure 6G).

## 3. Discussion

These studies have identified a duplicated leptin receptor, LepRA2, in rainbow trout and investigated the physiological responses of both leptin receptor paralogs to feed deprivation. In those vertebrates where a leptin receptor has been characterized, virtually all have only a single leptin receptor, including most teleost fishes where many genes have second copies due to genome duplication [16,28,29]. The LepRA2 paralog appears in the six other species of salmonids examined and they share a nucleotide sequence identity of 95–99% to that of Atlantic salmon LepRA2. There is greater divergence between paralogs as rainbow trout LepRA1 and LepRA2 amino acid sequences have 77.1% similarity. The primary sequence conservation with human LepR is low for both paralogs at 30.8% and 32.2% for trout LepRA1 and LepRA2, respectively, however the predicted receptor domains appear to be highly conserved. The phylogenetic analyses completed with the nucleotide sequences show two distinct clades formed for each of the leptin receptor paralogs in salmonids. Similarly, comparisons of amino acid sequences of leptin receptors show that the salmonid LepRA2 paralogs group separately from all LepRA1 and the LepRb paralogs of eel and arowana, suggesting the appearance of LepRA2 is likely due to the 4R salmonid specific genome duplication event [30,31]. Synteny analyses from another study proposed the eel LepRb was resultant from the teleost 3R genome duplication event and was subsequently lost in the teleost lineage [29]. While it remains to be evaluated fully, the retainment of the LepRb paralog is likely a similar case for the Asian arowana, with LepRa and LepRb protein sequences of the arowana and the eel grouping together respectively in the phylogenetic tree in Figure 2 and are notably separate from the salmonid LepRA2 node and LepRA1 of other teleost fish.

Teleost fishes are a diverse group of vertebrates and the responses of leptin and its receptors across taxa and even studies within a single species can vary [8,16]. The present study exposed rainbow trout to two weeks of feed deprivation to examine the response of leptin and the LepR paralogs. The feed deprived fish exhibited significantly lower body indices including body weight, length, blood glucose, HSI, and VSI, thus displaying the characteristic catabolic state induced from fasting. Interestingly, plasma leptin decreased with feed deprivation at one week and levels remained lower than fed fish at two weeks, albeit not significant at the latter timepoint (Figure 5F). Previous studies have shown mixed responses of leptin to fasting in rainbow trout. Fasting from one to four weeks elicited an increase of plasma leptin and the levels declined rapidly upon refeeding in *O. mykiss* [9,33]. Plasma leptin was higher in rainbow trout fed a restricted diet of 25% of controls for eight weeks [34]. An additional study analyzing changes in plasma leptin between two lines of trout with either high or low muscle adiposity suggests that nutritional plane (i.e., size of lipid depots) affects the leptin response [35]. In that study no changes in plasma leptin were measured in the high fat line while leptin levels decreased significantly in lean fish after two and four weeks of fasting. The rainbow trout used in the present study were smaller (~50 g) and contained relatively low lipid stores. Therefore, they are more likely to resemble the lean line of rainbow trout, which may be the basis for the decreased plasma leptin we observed. Additionally, long-term fasting of 4 months did not result in any significant plasma leptin or liver *lepa1* mRNA differences between fed and fasted rainbow trout [36]. We also did not detect any changes in hepatic *lepa1* mRNA over the two weeks of the study. Similarly, no changes in *lepa1* mRNA were recorded after four weeks of fasting in Atlantic salmon, but at seven weeks there was nearly a 20-fold increase in *lepa1* levels and a corresponding increase plasma leptin [37].

Responses of *lepa* mRNA to fasting are similarly inconsistent between studies and fish species (reviewed in [3,8]). It was suggested that environmental conditions such as temperature or photoperiod, and seasonal growth phases where energy utilization changes, may be a reason for variation observed in rainbow trout leptin responses [35]. It remains unclear why leptin can be differentially regulated with exposure to feed deprivation in rainbow trout and other fishes, but teleosts are a large and diverse group of vertebrates and it is likely that the actions of leptin are more complex than simply governing appetite [8]. Recent research suggests that leptin may act as a stress hormone during such catabolic events to aid in restoration of energy homeostasis (reviewed in [3]). Levels of *lepa* mRNA were stimulated by glucose and epinephrine injections in tilapia and it was proposed that leptin, in concert with classic stress hormones, may act to fine tune glucose during a stress response [6]. Perhaps the magnitude and duration of the stress response elicited from feed deprivation is important in understanding the wide-ranging responses of leptin. Additionally, there appears to be an important relationship between leptin, insulin, and glucose that requires further investigation in fishes. The existence of a leptin-insulin axis has been identified in tilapia, suggesting a conserved axis from mammals for the maintenance of glycemia during different metabolic states [38]. Furthermore, LepR mutant zebrafish exhibited no feeding phenotype, increased number of β-cells and insulin mRNA, and showed the hormone is primarily linked to glucose homeostasis [39].

A range of tissues were analyzed for mRNA abundance of the *lepa* and *lepr* paralogs in rainbow trout. *lepa1* and *lepa2* mRNA levels were highest in the liver, which is similar to previous reports in rainbow trout and other fishes [3,25,37,40,41]. While levels of *lepr* mRNA transcripts were detected in all tissues examined, there was tissue specific expression of the leptin receptor paralogs (Figure 4A). Interestingly, *lepra1* expression was low the liver while *lepra2* was expressed over 17× higher in this tissue. Likewise, Atlantic salmon also show tissue specific abundance of leptin receptor paralogs, including higher levels of *lepra2* in the liver and *lepra1* in the muscle, brain, and gills [30]. Both receptors exhibited substantial amounts mRNA levels in rainbow trout ovaries, however, *lepra1* was highest in this tissue at over 50× higher than *lepra2*. This could suggest that the *lepra1* paralog may play a greater role in reproduction or sexual maturation, both processes to which leptin has been implicated (reviewed in [42,43]). Although we did not evaluate *lepb* in the current paper, studies have shown that in some fishes the *lepb* paralog is highly expressed in the ovaries and brain compared to *lepa* (zebrafish, [44]; orange-spotted grouper, [45]; tongue sole, [46]). It is conceivable that for the case of rainbow trout where there are duplicated receptors, the *lepb* paralog along with *lepra1* may function together in more of a reproductive capacity or have a more specialized function. Interestingly, Atlantic salmon show increasing *lepra1* expression with maturation and both leptin receptor paralogs showed comparable levels in ovarian tissue of immature fish [30]. Additionally, Angotzi et al. suggested the preferred ligand/receptor combination for the brain could be LepA1/LepRA1 and LepB1-B2/LepRA1, while LepA1/LepRA2 interaction is favored in the liver [30]. Nevertheless, this aspect of leptin biology and the association between leptin paralogs and receptors requires further investigation.

While duplicated receptors have been characterized in Atlantic salmon [30] and European eel [29], the LepRA1 long form receptor along with truncated versions have been described in rainbow trout (LepRL, [26,32]), Atlantic salmon [25], European sea bass [24], and crucian carp [23]. The current study evaluated levels of duplicated leptin receptors, *lepra1* and *lepra2*, in the liver, muscle, and fat tissues from feed deprived rainbow trout. Low levels of *lepra1* were detected in the liver and did not change over the course of the study, however, *lepra2* was significantly higher than controls by one week of fasting and remained elevated at two weeks. Leptin receptors previously have shown to be regulated in the liver with fasting in fish. Hepatic *lepr* increased within one day of feed deprivation and remained elevated for three weeks in tilapia [10] and mRNA levels of hepatic *leprS3*, a short alternatively spliced leptin receptor variant, increased after 2 weeks of fasting in rainbow trout [26]. Although, no changes were observed in either LepR paralog with long-term fasting of four months in eel [29]. Interestingly, rainbow trout *lepra2* did not respond to fasting in both muscle and fat. This along with the low levels hepatic *lepra1* suggests the catabolic actions of leptin at the liver are through the *lepra2* paralog. Expression of *lepra2* was highest in the liver in immature female Atlantic salmon while no changes in hepatic receptors were observed between fish fed every day and fish that were on a restricted diet and only fed 3 days per week [30]. The absence of a hepatic *lepra2* response may have been due to the immature state of the salmon and/or that they were not completely fasted. They did observe a significant increase in brain *lepra1* and *lepa1* in fish on the partial diet [30]. In the current study, brain levels of the receptors were not measured in response to metabolic state, however, we did detect both LepR paralogs in the brain, hypothalamus, and pituitary. Regulation of leptin and its receptors in this region is not surprising due to the conserved role the brain plays in controlling food intake [47].

White muscle *lepra1* levels were 3-fold higher after one and two weeks of fasting, while transcripts in adipose tissue concurrently were suppressed at both timepoints. This suggests that in this feed deprived state, leptin is prioritizing the mobilization of energy substrates from white muscle via *lepra1* and through *lepra2* at the liver. In rainbow trout and most other fishes, leptin appears to primarily mobilize carbohydrates (*O. mykiss*, [48]; *O. mossambicus*; [49], reviewed in [3]), however, there is evidence in some fishes that leptin may also regulate lipids (*Carassius auratus*, [50]; *Pelteobagrus fulvidraco*, [51]; *Ctenopharyngodon idellus*, [52]). Glycogenolytic effects of LepA have been observed in rainbow trout with increased plasma glucose and reduced liver glycogen [48], although there is also indication that trout adipocytes can express and secrete leptin [34]. Rainbow trout that were selected for having high muscle adiposity had lower hypothalamic *leprl* (*lepra1*) expression than lean trout [53]. Although tissue glycogen and lipids were not quantified, the current study shows that amid transcripts of both receptor paralogs, muscle, hepatic, and adipose tissues were all regulated with exposure to feed deprivation. This implicates leptin actions on all three of these key metabolic tissues during a fasted, stressed state.

In summary, we have identified and characterized a duplicated leptin receptor, LepRA2, in rainbow trout. Evolutionary analyses show that the appearance of the LepRA2 paralog in trout and other salmonids was likely due to the 4R salmonid specific genome duplication event. The two leptin receptor paralogs identified in rainbow trout are differentially expressed across tissues and under catabolic conditions. During fasting leptin is likely acting to promote energy mobilization in the muscle through *lepra1* and in the liver through *lepra2* in rainbow trout. As we gain understanding about leptin and its receptors in fishes and other ectotherms, the pleiotropic behavior of this hormone becomes more apparent. While regulating appetite is a notable function of leptin, it is clear that through the tissue specific actions of its receptors the hormone can impact many other aspects of life history in fishes.

## 4. Materials and Methods

All procedures and research were approved and performed in accordance with the relevant guidelines and regulations by the Institutional Animal Care and Use Committee at the National Center for Cool and Cold Water Aquaculture (NCCCWA, protocol #164). Rainbow trout were maintained in either a flow-through tank or partial re-use system (temperature 12.5–13.5 °C, ambient photoperiod) and provided a commercially available feed (Zeigler Finfish G, Gardners, PA, USA; 42% protein, 16% fat).

### 4.1. Phylogenetic Analyses and Sequence Alignments

The rainbow trout genome (*O. mykiss*, accession # GCA_013265735.3) and all protein and nucleotide sequences for evolutionary analyses were retrieved from Genbank on the NCBI website ([54]; Bethesda, MD, USA). Published and predicted sequences for leptin receptors from salmonid fish were evaluated for homology. The *lepra2* sequence from Atlantic salmon (accession # JX131307, [30]) was compared to all similar salmonid sequences available on the Genbank database using BLAST [54]. For phylogenetic trees, all analyses were performed with MEGA X, where nucleotide and amino acid sequences of leptin receptors were aligned with Muscle and phylogenetic trees were created with the Maximum-likelihood method with default settings and 1000 bootstrap replicates [55]. An additional amino acid sequence alignment of the leptin receptors was performed using Clustal Omega (European Molecular Biology Laboratory, EMBL-EBI, Hinxton, Cambridgeshire, UK [56]) and analyzed with Jalview (version 2.11.1.3 [57]). The alignment conservation scores assigned by Jalview reflect the physical and chemical properties of each column in the alignment, with higher values indicating higher sequence conservation. The presence of conserved peptide signaling domains were predicted using the InterPro database and InterProScan (version 86.0; EMBL-EBI, Hinxton, Cambridgeshire, UK [58]).

### 4.2. Tissue Distribution

Adult female rainbow trout (1.7 ± 0.08 kg mean body weight, BW) that were approximately 21 months post-hatch were given a lethal dose (300 mg/L) of tricaine methanesulfonate (MS-222; Pentair Aquatic Eco-Systems, Apopka, FL, USA). Brain, distal kidney, fat, gill, heart, head kidney, hypothalamus, liver, ovary, pituitary, pyloric caeca, posterior intestine, red muscle, spleen, stomach, and white muscle were excised from the fish (*n* = 5). Tissues (~100 mg or less) were placed in 1 mL of RNAlater (Ambion Inc., Austin, TX, USA), kept overnight at 4 °C, and then stored at −20 °C until extractions.

### 4.3. Feed Deprivation Study

Rainbow trout (46.2 ± 5.7 g mean BW) were stocked 14 fish per tank into six 150-L tanks and were allowed to acclimate for one week, during which feed was provided at 2% of tank biomass using automated feeders (Arvotec, Finland). Three tanks were randomly assigned as fed controls and were fed 2% biomass/day and three tanks were fasted for two weeks. Body weights and lengths of all fish were recorded at the beginning of the study and at each timepoint. Control tanks were fasted for 24 h prior to sampling. Four fish from each tank were sampled at each timepoint, except at T = 0 where two fish from each of the six tanks were sampled (*n* = 12/treatment/timepoint). Fish were euthanized with a lethal dose of MS-222, length (cm) and weight (g) were recorded, and blood was collected from caudal vasculature using heparinized syringes. Blood glucose measurements were immediately analyzed using a Prodigy AutoCode glucometer (Prodigy Diabetes Care, LLC, Charlotte, NC, USA). Whole liver tissue was removed and weighed to obtain the hepatosomatic index (HSI; (liver weight/total body weight) × 100). Similarly, all the viscera were removed and weighed to calculate the viserosomatic index (VSI; (viscera weight/total body weight) × 100). Liver, white muscle, and fat tissues were collected (~100 mg) and placed in 1 mL of RNAlater, kept overnight at 4 °C, and then stored at −20 °C until extractions. Blood samples were spun at 3000× *g* for 7 min to collect plasma and stored at −20 °C.

### 4.4. RNA Isolation and Quantitative Real-Time PCR

Total RNA was extracted from tissues with 1 mL of Tri-Reagent (Molecular Research Center, Cincinnati, OH, USA) following the manufacturers protocol. RNA quality was assessed by presence of 18S and 28S ribosomal RNA bands using gel electrophoresis, and then quantified by absorbance OD 260:280 ratio using a Nanodrop 2000c spectrophotometer (Thermo Scientific, Waltham, MA, USA). Total RNA (≤ 1 µg) was DNase treated (Promega RQ1 RNase-Free DNase, Madison, WI, USA) and was used in a cDNA synthesis reaction via reverse transcription following the manufacturer’s instructions (Promega M-MLV Reverse Transcriptase). mRNA levels of *lepra1*, *lepra2*, *lepa1*, *lepa2*, and *ef-1α* (elongation factor 1 alpha) were determined by qPCR using gene-specific primers (Table 1).

Primer pairs were created with Primer-3 and BLAST on NCBI [59] and were designed to span into the transmembrane domain to ensure specificity to the long form receptor and not truncated forms or binding proteins. Primer pairs were compared to each other with BLAST to ensure no complementarity. *lepa1* and *lepa2* primers sequences were obtained from existing literature [60]. All reactions were run in triplicate and performed on a QuantStudio 5 Real-Time PCR System (Applied Biosystems, Foster City, CA, USA), with Applied Biosystems SYBR Green qPCR master mix, using 1.5 µM primers, and 2 µL of 1:6 diluted cDNA in a total reaction volume of 10 µL. The cycling parameters were 95 °C for 10 min followed by 40 cycles of 95 °C for 30 s and 60 °C for 1 min. A dissociation melt curve step at the end was performed to verify a single PCR product. The absence of genomic DNA contamination was confirmed using water (No Template Control; NTC) and DNase treated RNA with no reverse transcriptase enzyme (No-Amplification Control; NAC) as negative controls. Cycle threshold (Ct) values for samples were transformed using a standard curve of serially diluted pooled cDNA versus Ct values (R^2^ = 0.96–0.99). Samples were then normalized to reflect the amount of template cDNA per ng total RNA loaded into each reaction (cDNA/ng total RNA) and samples were also normalized to the expression levels of *ef-1α* RNA as a secondary control to validate the normalization method (data not presented). The expression of *ef-1α* has previously been validated as a stable reference gene in liver and white muscle our previous rainbow trout feed deprivation study [61]. The values are expressed as fold change relative to the mean of the initial baseline group as indicated in the figure legends.

### 4.5. Plasma Leptin Measurement

Plasma leptin was measured with a commercially available salmon leptin ELISA kit (Catalog # MBS935480; MyBioSource, Inc., San Diego, CA, USA). The assay was performed according to the manufacturers protocol. Plasma was diluted 1:75 or 1:200 in ultrapure water, followed by a 1:10 dilution with sample diluent. All samples and standards were run in duplicate. Optical density (OD) values were measured at 450 nm using a microplate reader (Varioskan, Thermo Scientific, Waltham, MA, USA). Readings at 570 nm and blank well ODs were subtracted from all wells to correct for background absorbance. Pooled plasma samples were run on each plate for interassay normalization. Adjusted OD values were analyzed using non-linear regression and GraphPad Prism 8 (GraphPad, La Jolla, CA, USA) and were interpolated from a sigmoidal curve generated from standards on each plate.

### 4.6. Statistical Analyses

The data were analyzed by two-way ANOVA (treatment x time) and were analyzed for significance at each time point with Fisher’s Least Significant Difference (LSD) test and also differences over time from control groups within treatments with Dunnett’s post-hoc. All analyses were performed using GraphPad Prism 8. The level set for statistical significance for all analyses was *p* < 0.05 and data are shown as mean values ± SEM.

## Figures and Tables

**Table 1:** List of primer sets used for qPCR in rainbow trout.

Gene	Accession #	Forward (5′-3′)	Reverse (5′-3′)
*lepra1*	JX878485	TCATTTCTATGACACTGAGTACGA	TCAGAAGCATGTAGGCAGCA
*lepra2*	XM_021599667	ACAGATGGAGAAGGAGAGCC	GGACAATGGCAAGGAAAGCG
*lepa1*	AB354909	GGTGATTAGGATCAAAAAGCTGGA	GACGAGCAGTAGGTCCTGGTAGAA
*lepa2*	JX123129	TGGGAATCAAAAAGCTCCCTTCCTCTT	GCCTCCTATAGGCTGGTCTCCTGCA
*ef-1α*	AF498320	CATTGACAAGAGAACCATTGA	CCTTCAGCTTGTCCAGCAC

*lepra1*: Leptin Receptor A1; *lepra2*: Leptin Receptor A2; *lepa1*: Leptin A1; *lepa2*: Leptin A2; *ef-1α*: Elongation factor 1 alpha.

## Data Availability

Data from this study will be provided upon request.
